# A Nationwide Enumeration of the Surgical Workforce, its Production and Disparities in Operative Productivity in Liberia

**DOI:** 10.1007/s00268-021-06379-8

**Published:** 2021-11-27

**Authors:** H. A. Adde, A. J. van Duinen, L. M. Sherman, B. C. Andrews, Ø. Salvesen, N. K. Dunbar, A. J. Bleah, T. G. Weiser, H. A. Bolkan

**Affiliations:** 1 Department of Clinical and Molecular Medicine, Faculty of Medicine and Health Sciences, NTNU–Norwegian University of Science and Technology, Post Box 8905, 7491 Trondheim, Norway; 2 Department of Surgery, St Olav’s Hospital, Trondheim University Hospital, Trondheim, Norway; 3 A. M. Dogliotti College of Medicine, University of Liberia, Monrovia, Liberia; 4 Liberia College of Physicians and Surgeons, Monrovia, Liberia; 5 Department of Public Health and Nursing, Faculty of Medicine and Health Sciences, NTNU–Norwegian University of Science and Technology, Trondheim, Norway; 6 Research Unit, Ministry of Health, Monrovia, Liberia; 7 Esther and Jereline Medical Center, Ganta, Liberia; 8 Department of Surgery, Stanford University, Stanford, USA; 9 Stanford-Surgery Policy Improvement Research and Education Center, Department of Surgery, Stanford University, Palo Alto, USA; 10 Department of Clinical Surgery, University of Edinburgh, Edinburgh, UK

## Abstract

**Background:**

Any health care system that strives to deliver good health and well-being to its population relies on a trained workforce. The aim of this study was to enumerate surgical provider density, describe operative productivity and assess the association between key surgical system characteristics and surgical provider productivity in Liberia.

**Methods:**

A nationwide survey of operation theatre logbooks, available human resources and facility infrastructure was conducted in 2018. Surgical providers were counted, and their productivity was calculated based on operative numbers and full-time equivalent positions.

**Results:**

A total of 286 surgical providers were counted, of whom 67 were accredited specialists. This translated into a national density of 1.6 specialist providers per 100,000 population. Non-specialist physicians performed 58.3 percent (3607 of 6188) of all operations. Overall, surgical providers performed a median of 1.0 (IQR 0.5–2.7) operation per week, and there were large disparities in operative productivity within the workforce. Most operations (5483 of 6188) were categorized as essential, and each surgical provider performed a median of 2.0 (IQR 1.0–5.0) different types of essential procedures. Surgical providers who performed 7–14 different types of essential procedures were more than eight times as productive as providers who performed 0–1 essential procedure (operative productivity ratio = 8.66, 95% CI 6.27–11.97, *P* < 0.001).

**Conclusion:**

The Liberian health care system struggles with an alarming combination of few surgical providers and low provider productivity. Disaggregated data can provide a high-resolution picture of local challenges that can lead to local solutions.

## Introduction

The global burden of disease is shifting towards non-communicable diseases and injuries [1, 2]. All-cause mortality will not decrease at the same pace as in previous decades unless major efforts are implemented to reorient health systems [2]. Affordable surgical services should be an essential part of any health care system [3, 4]. A trained surgical workforce is key to deliver safe surgical services [3, 4], but the global workforce of specialist surgical providers is maldistributed and to a large extent inadequate to cover population needs, especially in low-income countries [3, 5].

The density of specialist surgical providers has been described as a key metric to benchmark and monitor development worldwide [3]. To complement global metrics, subnational analysis has been highlighted as an important tool to reveal in-country challenges [6, 7]. Operative productivity is a measure that has been used to describe surgical systems both at the provider level [8] and at the facility level [9]. Although surgical facilities are often highlighted as the nucleus of surgical systems [6], granular data on surgical provider specialization, geographic distribution and productivity can add valuable information [10]. However, there is no consensus on how to report on these detailed variables and limited knowledge about the relationship between them in settings where resources are sparse.

Liberia is located on the shoreline of West-Africa and is ranked 175 out of 189 nations on the United Nations Human Development Index [11]. More than a decade of civil war and the more recent Ebola virus outbreak has weakened the health system [12], and surgical volumes are critically low [13, 14]. The country has one medical school that is located in the capital Monrovia [15], and the annual output of medical graduates averages 25 doctors per year [16]. To strengthen surgical services, the Liberia College of Physicians and Surgeons was established in 2013, but the postgraduate program is struggling with deficits in human resources to teach and supervise trainees as well as infrastructural shortages [16].

There is a need to strengthen the surgical sector if universal health coverage is to be achieved in Liberia. A baseline assessment of the surgical system, including its workforce, is a necessary first step before launching strengthening initiatives [6, 17]. Consequently, this study aimed to enumerate surgical provider density, describe operative productivity and assess the association between key surgical system characteristics and surgical provider productivity.

## Material and methods

### Data collection

A nationwide survey of surgical activity, human resources and infrastructure in Liberia was conducted between 20 September and 8 November 2018. All healthcare facilities that performed surgical procedures requiring general, regional or local anaesthesia within an operating theatre the year prior to the data collection were eligible for inclusion. In each facility, structured interviews were conducted to collect information on surgical providers. The Lancet Commission on Global Surgery hospital assessment tool was used to quantify facility infrastructure and equipment. Operation theatre logbooks were reviewed, and a sample of four preselected months from the logbooks (October, January, April and July) was transcribed into a Microsoft Excel dataset (Microsoft, Redmond, Washington, USA). The data collection has been described in detail elsewhere [14].

### Definitions

A surgical provider was defined as any provider listed as the main operator in the 4-month sample from the operation theatre logbooks. All surgical providers were categorized according to specialization level: accredited specialist, specialist program resident, non-specialist physician or non-physician (physician assistant, midwife or nurse). The provision of safe surgery was defined in-line with previous studies [18, 19] and included the availability of eight infrastructural items at the facility: pulse oximeter, adult bag mask, oxygen, suction, intravenous fluids, sterile gloves, skin preparation solution and a functioning sterilizer.

All operations requiring general, regional or local anaesthesia within an operating theatre were defined as a surgical procedure [20]. A surgical procedure listed as especially cost-effective by the third edition of the World Bank Disease Control Priorities was defined as essential [4]. Geographic areas were categorized according to population poverty levels (proportion of population living in absolute poverty), as defined by the Liberian Household Income and Expenditure Survey [21].

### Analysis

Surgical provider density was calculated per 100,000 population [3]. Population numbers were extracted from the Liberian Household Income and Expenditure Survey [21] and scaled up using the population growth rate from the latest population census [22]. Calculating essential surgical procedures as a proportion of total surgical numbers has previously been proposed as a method to describe the impact level of surgical volumes [23], and this method was applied. Also, the number of different essential procedures performed was counted for each surgical provider, giving a range of essential surgical procedures performed [14]. A score that combines the presence of multiple infrastructure and equipment items [14] was used to describe the level of infrastructure available to each surgical provider in the facility they worked. Surgical providers that practiced in several facilities were allocated to the facility where they spent most time.

The basic unit for calculating operative productivity was the surgical provider, and all types of operations performed were included. Surgical provider productivity was defined as the total number of operations performed as first operator per full-time equivalent position per week, as described by a previous study [8]. The length and the size (full time or part time) of a surgical provider working position over the study period were combined into one full-time equivalent position measure. Consequently, provider productivity was adjusted for working position length and size, leaving a measure of weekly performed operations per surgical provider. The operations and working position for a provider working in multiple facilities were combined into one productivity measure for that provider.

A mixed Poisson regression model was built in RStudio version 1.3.1093 [24] with lme4 [25] to perform linear mixed analysis of the association between surgical system characteristics and surgical provider productivity. The basic unit included in the regression model was the surgical providers, and these were described by individual and facility characteristics. The facility characteristics were shared for providers working in the same surgical facility. One regression model was used to assess the association between variables at the individual level and at the facility level and surgical provider productivity. Surgical system variables were included as fixed effects and surgical provider identification as a random effect. The effect measure is an operative productivity ratio (OPR). An OPR of two implies a twofold increase in productivity compared with the reference. A multivariable model was run adjusting for surgical provider full-time equivalent position, number of facility beds and number of facility operation theatres. The full-time equivalent position was included as an offset variable.

### Ethical considerations

The Institution Review Board, University of Liberia granted ethical clearance (number FWA00004982) and the Regional Committee for Medical and Health Research Ethics in central Norway exempted this study from review (number 2018/1008).

## Results

### The surgical workforce

**Fig. 1:**
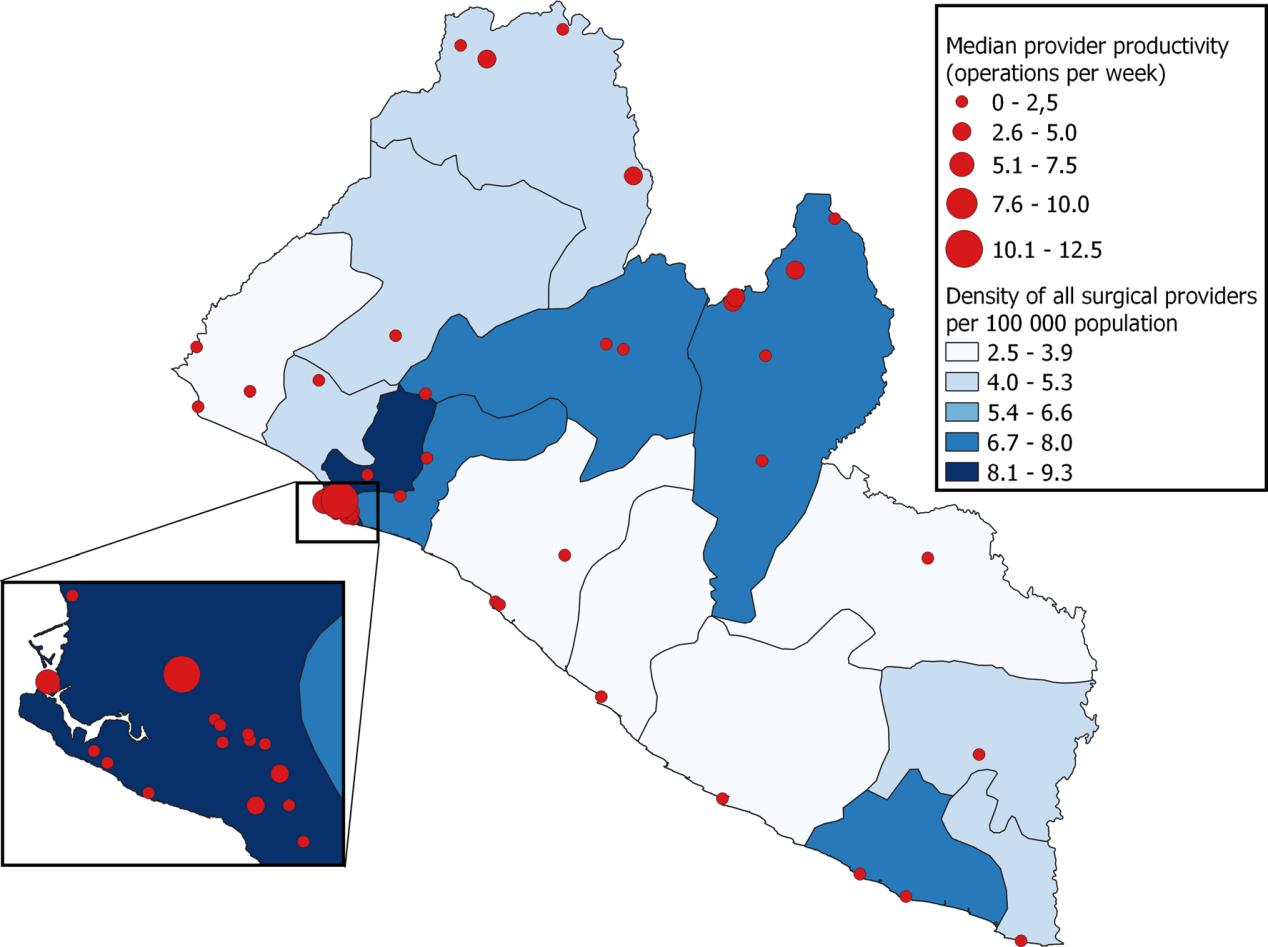
Geographic distribution of the surgical workforce and surgical provider productivity

**Fig. 2:**
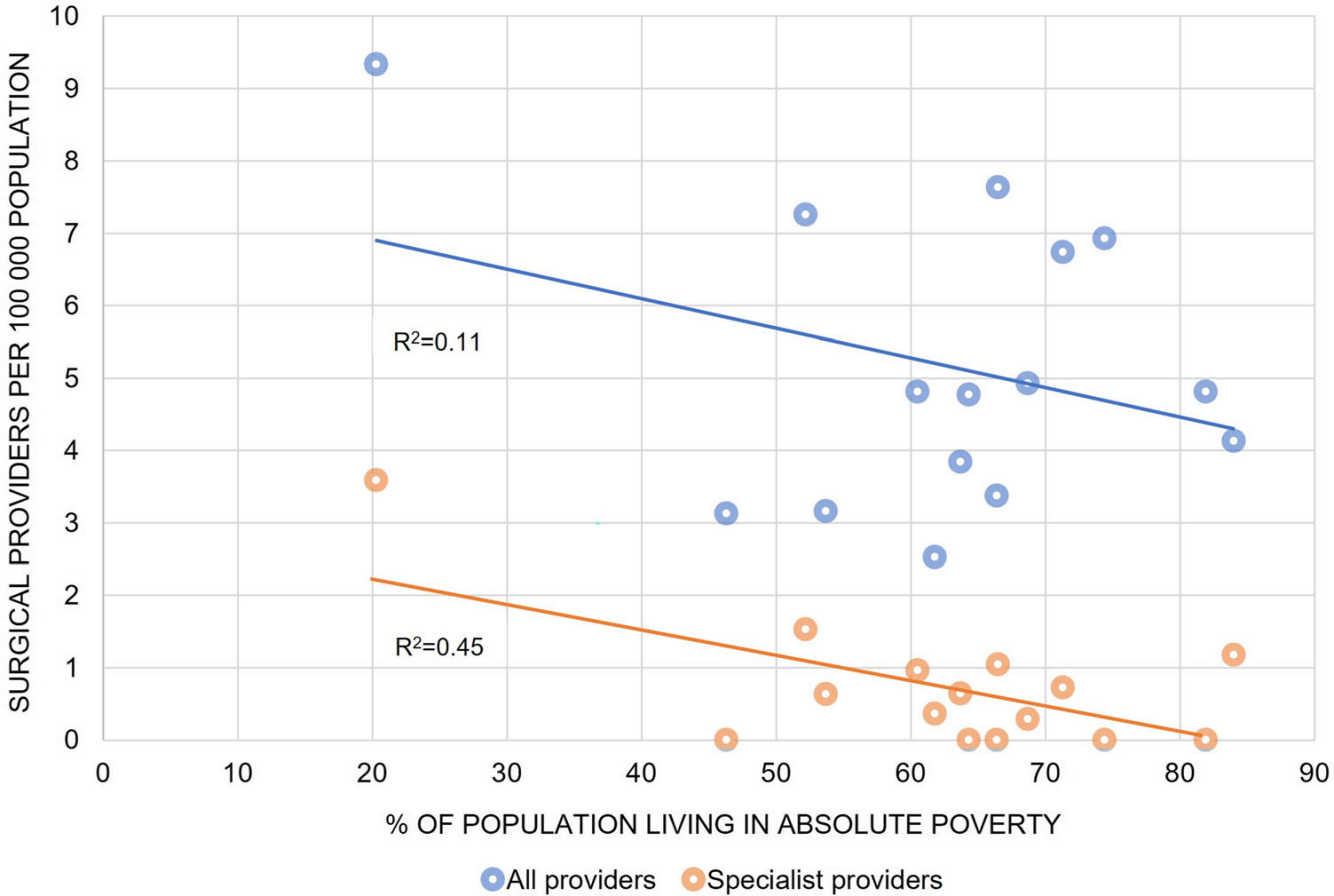
Density of surgical providers and proportion of people living in absolute poverty. Each data point represents one of the fifteen counties (administrative areas) in Liberia

A total of 286 surgical providers were registered in the operation theatre logbook of 4-month sample, and 67 of these were accredited specialists (Table 1). Of the specialists, 48 worked in the capital area. Nineteen surgical providers were practicing in more than one facility. A major part of the surgical workforce were non-specialist physicians (152 of 286), and these performed 58.3 percent of all operations (3607 of 6188). The national density of all surgical providers was 6.7 per 100,000 population, and the density of specialists, including anaesthesiologists, was 1.6 per 100,000 population. There were three specialist anaesthesiologists working in three different facilities at the time of the data collection. Areas with higher poverty had lower specialist density (0.7 per 100,000 population) compared with areas with lower poverty (3.6 per 100,000 population). Between all 15 counties (administrative areas), the specialist density ranged from 0 to 3.6 per 100,000 population. Montserrado County, where the capital Monrovia is located, had the highest density of surgical providers (Fig. 1). Counties with high population poverty had the lowest density of surgical providers (Fig. 2).

**Table 1:** Density and characteristics of surgical providers by specialization level

	Specialists	Residents	Non-specialist physicians	Non-physicians	Total
	*n* = 67	*n* = 48	*n* = 152	*n* = 19	*n* = 286
Provider characteristics^a^					
*Working position*					
Full time	43 (64.2)	19 (39.6)	72 (47.4)	7 (36.8)	141 (49.3)
Part time	23 (34.3)	29 (60.4)	80 (52.6)	10 (52.6)	142 (49.7)
Missing	1 (1.5)	0 (0)	0 (0)	2 (10.5)	3 (1.1)
*Nationality*					
Liberian	32 (47.8)	46 (95.8)	123 (80.9)	18 (94.7)	219 (76.6)
Foreign	35 (52.2)	2 (4.2)	29 (19.1)	0 (0)	66 (23.1)
Missing	0 (0)	0 (0)	0 (0)	1 (5.3)	1 (0.4)
*Operations per week*^*b*^					
All providers	1.0 (0.5–2.8)	0.7 (0.3–2.4)	1.3 (0.5–2.8)	0.7 (0.3–1.4)	1.0 (0.5–2.7)
Area with lower poverty^c^	0.8 (0.5–3.1)	0.7 (0.3–1.3)	1.4 (0.5–3.0)	0.9 (0.6–1.9)	0.9 (0.5–2.5)
Area with intermediate poverty^d^	1.5 (0.8–3.1)	0.5 (0.3–1.0)	1.3 (0.5–3.1)	0.5 (0.2–1.2)	1.1 (0.4–3.0)
Area with higher poverty^e^	0.9 (0.5–1.7)	3.7 (0.7–5.6)	1.1 (0.2–2.2)	1.0 (0.3–2.3)	1.1 (0.3–2.4)
Surgical provider density^f^					
All providers	1.6	1.1	3.5	0.4	6.7
Area with lower poverty^c^	3.6	2.7	2.8	0.3	9.3
Area with intermediate poverty^d^	0.7	0.1	4.0	0.5	5.2
Area with higher poverty^e^	0.7	1.1	3.5	0.7	5.9
Surgical procedures					
*Four-month surgical volume*^*g*^					
All procedures	1631	807	3607	143	6188
Essential procedures	1269 (77.8)	748 (92.7)	3329 (92.3)	137 (95.8)	5483 (88.6)
Other procedures	362 (22.2)	59 (7.3)	278 (7.7)	6 (4.2)	705 (11.4)
*Range of essential procedures*^*b*^					
All procedures	3.0 (2.0–7.0)	2.0 (1.0–5.0)	2.0 (1.0–5.0)	1.0 (1.0–2.0)	2.0 (1.0–5.0)
Obstetrics/gynaecology	1.0 (0–2.0)	1.0 (0–2.0)	1.0 (1.0–2.0)	1.0 (1.0–1.0)	1.0 (1.0–2.0)
General surgery	2.0 (0–3.0)	0.5 (0–2.0)	1.0 (0–2.0)	0 (0–0)	1.0 (0–2.0)
Injury/orthopaedics	1.0 (0–2.0)	0 (0–1.0)	0 (0–1.0)	0 (0–0)	0 (0–1.0)
Area with lower poverty^c^	3.0 (2.0–7.0)	2.0 (1.0–5.0)	2.0 (1.0–4.0)	1.5 (0.3–2.0)	2.0 (1.0–5.0)
Area with intermediate poverty^d^	3.5 (1.0–6.3)	1.0 (1.0–2.0)	3.0 (1.0–6.0)	1.0 (1.0–2.0)	2.0 (1.0–5.3)
Area with higher poverty^e^	5.0 (1.5–6.5)	3.0 (1.0–5.0)	2.5 (1.0–6.3)	1.0 (0.5–2.0)	2.0 (1.0–5.0)

^a^Number (%); ^b^Median (IQR); ^c^Area where < 30% of people live in absolute poverty; ^d^Area where 30–70% of people live in absolute poverty; ^e^Area where > 70% of people live in absolute poverty; ^f^Density per 100,000 population; ^g^240 operations excluded due to unknown procedure name and/or surgical provider

### Operative productivity

**Fig. 3:**
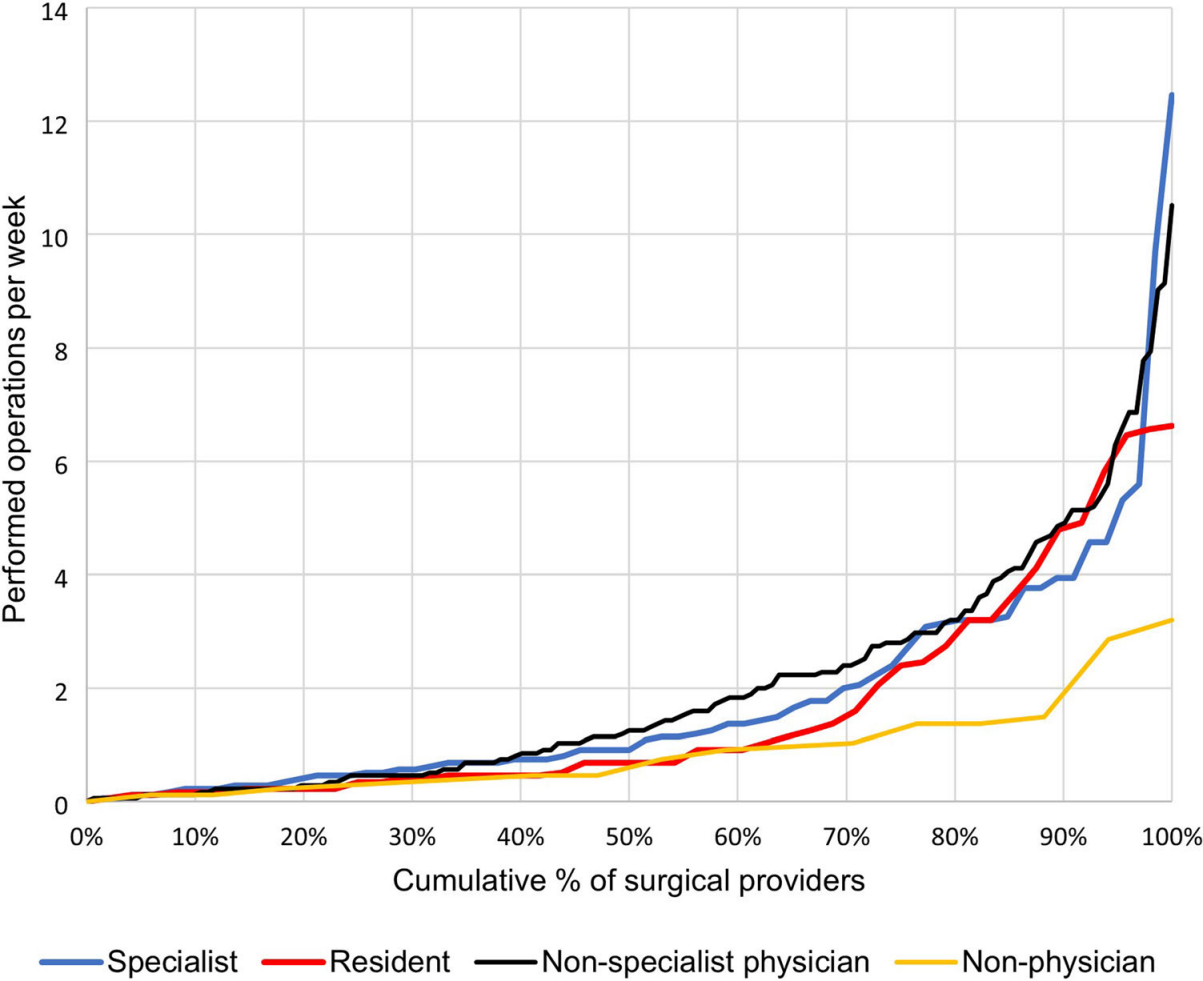
Cumulative percentage of surgical providers by cadre and their operative productivity

The median provider productivity was 1.0 (IQR 0.5–2.7) operation per week (Table 1), and there were large differences in operative productivity (Fig. 3). The most productive 10 percent (28 of 286) of the surgical workforce performed 36 percent (2237 of 6188) of the total surgical volume. Eighteen of the 28 highest performing providers were non-specialist physicians. The 28 most productive providers were spread across 19 different surgical facilities.

### Essential and other surgical procedures

Of all surgical procedures performed, 88.6 percent (5483 of 6188) were categorized as essential (Table 1). Specialists performed 77.8 percent (1269 of 1631) essential procedures compared with 92.3 percent (3329 of 3607) essential procedures performed by non-specialist physicians. Surgical providers performed a median of 2.0 (IQR 1.0–5.0) different types of essential procedures. Specialists working in areas with high population poverty performed a median of 5.0 (IQR 1.5–6.5) different types of essential procedures. Other surgical procedures constituted 11.4 percent of the total surgical volume (Table 1), and specialists performed a median of 2.0 (IQR 1.0–3.0) other procedures, while residents, non-specialist physicians, and non-physicians performed 1.0 (IQR 0–1.0), 0 (IQR 0–2.0) and 0 (IQR 0–0) other surgical procedures, respectively.

### Surgical system variables and their association with provider productivity

Surgical providers who performed 7–14 different essential procedures had an operative productivity ratio (OPR) 8.66 (95% CI 6.27–11.97, *P* < 0.001) times higher than providers who performed 0–1 essential procedures (Table 2). Surgical providers working in private non-profit facilities were almost three times as productive (OPR = 2.82, 95% CI 1.71–4.65, *P* < 0.001) as providers working in governmental facilities (Table 3). However, providers working in facilities with a better infrastructure score (165) did not have a significantly higher productivity (OPR = 1.17, 95% CI 0.73–1.88, *P* = 0.512). Shortages of certain safe surgery items like oxygen (OPR = 0.62, 95% CI 0.42–0.90, *P* = 0.012) and blood supply (OPR = 0.60, 95% CI 0.39–0.93, *P* = 0.020) seemed to be associated with provider productivity (Table 4).

**Table 2:** Association between surgical provider characteristics and operative productivity

	Surgical providers (*n*)^a^	Univariable model		Multivariable model	
		Operative productivity ratio (95% CI)	*P*	Operative productivity ratio (95% CI)	*P*
Range of essential procedures					
0–1	84	1.00 (reference)		1.00 (reference)	
2	59	2.67 (1.94–3.67)	< 0.001	2.81 (2.04–3.90)	< 0.001
3	38	4.24 (2.97–6.07)	< 0.001	4.19 (2.92–6.03)	< 0.001
4–6	53	5.49 (3.98–7.58)	< 0.001	4.49 (3.25–6.20)	< 0.001
7–14	49	14.20 (10.26–19.65)	< 0.001	8.66 (6.27–11.97)	< 0.001
Caesarean section					
Performing	206	1.00 (reference)		1.00 (reference)	
Not performing	77	0.37 (0.27–0.52)	< 0.001	0.45 (0.32–0.62)	< 0.001
Laparotomy					
Performing	85	1.00 (reference)		1.00 (reference)	
Not performing	198	0.31 (0.23–0.42)	< 0.001	0.41 (0.31–0.54)	< 0.001
Open fracture treatment					
Performing	11	1.00 (reference)		1.00 (reference)	
Not performing	272	0.34 (0.16–0.72)	0.005	0.44 (0.22–0.88)	0.021
Provider specialization					
Specialist	66	1.00 (reference)		1.00 (reference)	
Resident	48	0.57 (0.35–0.93)	0.024	0.96 (0.60–1.52)	0.854
Non-specialist physician	152	0.86 (0.60–1.25)	0.435	0.82 (0.57–1.19)	0.302
Non-physician	17	0.45 (0.22–0.90)	0.024	0.48 (0.25–0.93)	0.030
Provider nationality					
Liberian	218	1.00 (reference)		1.00 (reference)	
Foreign	65	1.70 (1.19–2.42)	0.004	1.62 (1.17–2.25)	0.004
Working position					
Full time	141	1.00 (reference)		1.00 (reference)	
Part time	142	0.45 (0.34–0.60)	< 0.001	1.09 (0.83–1.44)	0.545
Working area					
Area with lower poverty^b^	126	1.00 (reference)		1.00 (reference)	
Area with intermediate poverty^c^	113	0.94 (0.67–1.31)	0.697	0.77 (0.50–1.19)	0.244
Area with higher poverty^d^	44	0.92 (0.59–1.44)	0.716	0.83 (0.49–1.40)	0.487

^a^Three missing values on provider productivity; ^b^Area where < 30% of people live in absolute poverty; ^c^Area where 30–70% of people live in absolute poverty; ^d^Area where > 70% of people live in absolute poverty

**Table 3:** Association between facility characteristics and surgical provider operative productivity

	Surgical providers (*n*)^a^	Univariable model		Multivariable model	
		Operative productivity ratio (95% CI)	*P*	Operative productivity ratio (95% CI)	*P*
Facility owner					
Governmental	183	1.00 (reference)		1.00 (reference)	
Private non-profit	68	1.41 (0.99–2.03)	0.060	2.82 (1.71–4.65)	< 0.001
Private for-profit	32	0.99 (0.60–1.61)	0.956	1.44 (0.85–2.45)	0.178
Facility infrastructure score					
97–148	79	1.00 (reference)		1.00 (reference)	
149–157	90	0.99 (0.68–1.46)	0.952	1.23 (0.78–1.92)	0.371
158–164	42	1.57 (0.97–2.53)	0.068	1.29 (0.74–2.28)	0.372
165	72	0.83 (0.55–1.26)	0.378	1.17 (0.73–1.88)	0.512
Surgical providers per facility					
1–4	68	1.00 (reference)		1.00 (reference)	
5–11	78	0.74 (0.48–1.12)	0.153	0.68 (0.42–1.09)	0.107
12–17	72	0.83 (0.54–1.27)	0.386	0.79 (0.43–1.43)	0.429
18–37	65	0.65 (0.42–1.01)	0.056	0.74 (0.32–1.75)	0.500
Surgeon specialist^b^					
Present	133	1.00 (reference)		1.00 (reference)	
Not present	150	1.27 (0.94–1.73)	0.123	1.15 (0.76–1.74)	0.504
Ob/gyn specialist^b^					
Present	117	1.00 (reference)		1.00 (reference)	
Not present	166	1.40 (1.03–1.91)	0.032	1.22 (0.87–1.71)	0.252
Anaesthesiologist specialist^b^					
Present	55	1.00 (reference)		1.00 (reference)	
Not present	228	1.41 (0.96–2.09)	0.082	1.48 (0.71–3.11)	0.295

^a^Three missing values on provider productivity; ^b^At least one specialist available at the facility where the surgical provider works

**Table 4:** Association between available safe surgery items and surgical provider productivity

	Surgical providers (*n*)^a^	Univariable model		Multivariable model	
		Operative productivity ratio (95% CI)	*P*	Operative productivity ratio (95% CI)	*P*
Safe surgery items					
*Oxygen*					
Available	186	1.00 (reference)		1.00 (reference)	
Not available	97	0.87 (0.63–1.20)	0.401	0.62 (0.42–0.90)	0.012
*Pulse oximetry*					
Available	270	1.00 (reference)		1.00 (reference)	
Not available	13	0.96 (0.46–1.98)	0.904	1.34 (0.65–2.74)	0.431
*Sterilizer*					
Available	283	1.00 (reference)		1.00 (reference)	
Not available	0	–	–	–	–
*Skin preparation solution*					
Available	280	1.00 (reference)		1.00 (reference)	
Not available	3	0.29 (0.058–1.46)	0.133	0.18 (0.041–0.78)	0.022
*Sterile gloves*					
Available	278	1.00 (reference)		1.00 (reference)	
Not available	5	0.91 (0.28–2.92)	0.874	0.90 (0.31–2.65)	0.850
*Suction*					
Available	280	1.00 (reference)		1.00 (reference)	
Not available	3	0.74 (0.16–3.33)	0.691	0.61 (0.15–2.44)	0.487
*Adult mask bag*					
Available	283	1.00 (reference)		1.00 (reference)	
Not available	0	-	-	-	-
*Intravenous fluids*					
Available	239	1.00 (reference)		1.00 (reference)	
Not available	44	0.85 (0.56–1.29)	0.442	0.72 (0.47–1.11)	0.134
All safe surgery items					
Available	163	1.00 (reference)		1.00 (reference)	
Not available	120	0.91 (0.67–1.24)	0.543	0.73 (0.49–1.07)	0.110
Blood available < 30 min					
Available	239	1.00 (reference)		1.00 (reference)	
Not available	44	0.82 (0.54–1.25)	0.360	0.60 (0.39–0.93)	0.020
Safe surgery checklist					
Utilized	117	1.00 (reference)		1.00 (reference)	
Not utilized	166	0.99 (0.72–1.35)	0.935	0.99 (0.67–1.48)	0.975

^a^Three missing values on provider productivity

## Discussion

This study aimed to describe the surgical workforce, its production and variables associated with surgical provider productivity in Liberia. The density of specialist providers was 1.6 per 100,000 population, and 58 percent of all operations were performed by non-specialist physicians. Surgical providers performed a median of one operation per week, and there were large disparities in operative productivity within the surgical workforce.

Global targets recommend a minimum threshold of 20 surgical, anaesthetic and obstetric specialist physicians per 100,000 population by 2030 [3]; with three specialist anaesthesiologists, we identified a critically low density of surgical specialists in Liberia. Furthermore, the specialist workforce is maldistributed, with the lowest presence of specialists in areas where population poverty is high. To avoid increasing inequality in health, strategies to strengthen surgical human resources should not only focus on increasing the number of surgical providers, but also seek to distribute these more equally. Clear policies on workforce distribution can be an important tool to retain health workers in rural areas and counteract disparities in health [26].

Increasing the number of surgical providers is one of several strategies that should be explored if total surgical volume is to be increased effectively, especially in countries where population numbers are growing [27]. Surgical provider productivity is an important measure because increasing individual operative productivity is one way of boosting surgical volumes. Building surgical teams where specialized providers train and supervise more numerous non-specialist physicians who can deal with a larger volume of less complicated cases may increase the workforce productivity. Equally important, provider volume is known to be closely related to surgical outcomes [28, 29]. The present study highlighted major disparities in surgical provider productivity, and many providers are rarely entering the operating theatre. The explanation for low productivity is probably complex, as barriers to surgical care in low-resource settings include cultural, financial and structural elements [30]. Nonetheless, one striking aspect is the improved productivity among residents working in areas where the population poverty is higher. These areas all have fewer surgical providers, which might make it easier for the residents to access the operation theatre. The low productivity illustrates how in-depth descriptions can be a steppingstone for further investigations into underlying causes and thereby contribute to building solid fundaments for new policies.

The Lancet Commission on Global Surgery suggested to benchmark and monitor the surgical workforce through specialist provider density [3]. In Liberia, 58 percent of all operations are performed by non-specialist physicians, and these providers are the backbone of surgical services outside the capital area. Non-specialist physicians are known to be major contributors to surgical care in sub-Saharan Africa [31, 32], but their presence is currently not captured by global benchmarking metrics. This study highlights that non-specialist physicians can be major contributors to national surgical volumes, and they may also be part of the solution in making surgical services more available. Hence, their presence should be recognized, measured and monitored.

Total surgical volume is an important metric for global surgery benchmarking [3]. Differentiating priority levels for operations by categorizing them as essential or not has been suggested as the next step within surgical volume benchmarking [23]. Almost 90 percent of all operations in this study was categorized as essential, indicating a high population impact. However, the median surgical provider only performed two different types of essential procedures, most commonly caesarean section and hernia repair. A limited availability of essential procedures has also been described at the facility level in Liberia [14], implying that many essential operations are not being performed. This should be seen in the context of low surgical volumes [14], which is far below the Lancet Commission threshold [3], showing that 91 percent of the recommended surgical volume is unmet. Benchmarking metrics should not only include aggregated surgical volume, but also describe the availability of the separate essential procedures to provide a better understanding of the challenges and opportunities within the surgical system.

As global surgery develops, there is an increasing momentum to construct national surgical, obstetric and anaesthesia plans, and these plans should be based on national assessments of the surgical system, including the specialist workforce [17]. In Liberia, the operative productivity among specialists was low, and the difference in productivity between specialists and non-specialist physicians was small. This raises questions about the comparability of specialists across settings where surgical volumes are very different. It may be the case that specialists mainly supervise and act as support for less experienced first operators. However, if this was the case, one would expect providers working in facilities with specialists present to be more productive, but such a difference was not detected. Due to the immense heterogeneity between nations and the nature of their health systems, local metrics should reflect local circumstances [33]. A focus on specialists alone will in many low- and middle-income countries only describe a minor fraction of the full surgical workforce. Consequently, overarching global metrics should be disaggregated when used at the national level to set aspirational targets for national plans.

This study is to the best of our knowledge the most comprehensive assessment of the surgical workforce in Liberia to date, creating a foundation for recommendations (Textbox). However, some limitations should be considered when interpreting the results. Firstly, the operative characteristics rely on a 4-month sample, and although the sample scale up to 12 months correlated well with the actual count from all 12 months [14], there is a chance that some seasonal variation was missed. Furthermore, surgical providers were identified through the 4-month sample, and it is possible that surgeons practicing in-between these 4 months were missed. Additionally, only the first operator was counted, and the efforts from second operators were not captured. It may be the case that experienced providers mainly teach and supervise as second operator, and such efforts are not reflected in the individual operative productivity measure. Another limitation is that this study did not provide complementary information on anaesthesia capabilities that may influence operative productivity. Lastly, the functionality of safe surgery items was not systematically assessed through inspection, and some of these items may have been considered inadequate for its use if more detailed information had been obtained.

**Figure:**
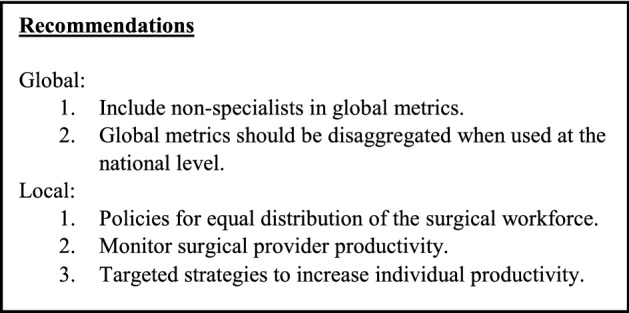
Textbox: Recommendations to strengthen reporting metrics and improve the surgical system globally and locally in Liberia

This study has highlighted a critical shortage of surgical providers in Liberia, and specialists are particularly deficient. Furthermore, most of the surgical workforce is rarely entering the operating theatre, which raises concerns about the quality of care. However, this challenge also offers an opportunity to boost surgical volumes if productivity can be lifted. Comprehensive training that allows providers to offer a broader range of essential procedures may also allow them to be more productive. This study also demonstrates that disaggregated data analysed at the lowest level of resolution can highlight challenges and opportunities and inform national planning. Such analysis can separate facility-level trends from provider-level trends and can lead to good targets at each level. The evolution of surgical system assessments and reporting metrics will be key if the global surgery movement is to keep its course towards universal health coverage.
